# Daily and Nondaily E‐Cigarette Use in Relation to Cigarette Consumption Among Current Smokers

**DOI:** 10.1111/crj.70219

**Published:** 2026-07-31

**Authors:** Yusuff Adebayo Adebisi

**Affiliations:** ^1^ College of Social Sciences University of Glasgow Glasgow UK; ^2^ Department of Clinical & Experimental Medicine University of Catania Catania Italy

**Keywords:** cigarette consumption, cross‐sectional study, dual use, e‐cigarettes, Scottish Health Survey, smoking intensity

## Abstract

**Background:**

Dual use of e‐cigarettes and conventional cigarettes is the most common pattern of e‐cigarette use among adults who smoke. A central unresolved question is whether e‐cigarettes partially displace cigarette consumption among dual users or supplement largely unchanged smoking, and whether any difference depends on the frequency of e‐cigarette use. Population‐based evidence examining smoking intensity and stratifying dual users by frequency of e‐cigarette use remains limited.

**Methods:**

A pooled cross‐sectional analysis was conducted using five waves (2017, 2018, 2019, 2021 and 2022) of the Scottish Health Survey, a nationally representative household survey. The analytic sample comprised 3150 current cigarette smokers aged 16 years and over, including 2605 cigarette‐only smokers and 545 dual users of cigarettes and e‐cigarettes. Two outcomes were examined: average number of cigarettes smoked per day and smoking intensity classified as light (< 10/day), moderate (10 to < 20/day) or heavy (≥ 20/day). Associations were estimated using multivariable linear regression, generalised ordered logit models and quantile regression, adjusting for age, sex, area deprivation, ethnicity, educational attainment and survey year. An additional analysis stratified dual users by frequency of e‐cigarette use as daily, weekly nondaily or less‐than‐weekly users. Postestimation contrasts compared the frequency groups directly.

**Results:**

Dual users smoked fewer cigarettes per day than cigarette‐only smokers (mean: 11.28 [SD: 7.07] vs. 12.65 [SD: 7.94]; *p* = 0.0002). After full adjustment, this corresponded to 1.10 fewer cigarettes per day (β = −1.10; 95% CI: −1.73 to −0.47; *p* = 0.001). Dual users also had 20% lower odds of being in a higher smoking‐intensity category (OR = 0.80; 95% CI: 0.67 to 0.96; *p* = 0.014). The difference in cigarette consumption was larger at the upper end of the distribution, reaching approximately two fewer cigarettes per day at the 90th percentile (β = −1.99; 95% CI: −3.32 to −0.65; *p* = 0.004). In frequency‐specific analyses, only daily e‐cigarette users differed from cigarette‐only smokers. Daily e‐cigarette users smoked a mean of 9.90 cigarettes per day compared with 12.65 among cigarette‐only smokers (adjusted β = −2.46; 95% CI: −3.32 to −1.59; *p* < 0.001) and had lower odds of being in a higher smoking‐intensity category (OR = 0.57; 95% CI: 0.44 to 0.74; *p* < 0.001). Neither less‐than‐weekly nor weekly nondaily e‐cigarette use was associated with lower cigarette consumption. Formal contrasts showed that daily e‐cigarette users smoked 2.21 fewer cigarettes per day than less‐than‐weekly users (*p* = 0.010) and 2.23 fewer than weekly nondaily users (*p* = 0.001).

**Conclusions:**

Concurrent e‐cigarette use was associated with modestly lower cigarette consumption among current smokers, but only among daily e‐cigarette users, who still smoked about 10 cigarettes per day. This pattern may reflect partial cigarette displacement, selection or reverse causation, whereas nondaily e‐cigarette use was more consistent with supplementary use. Dual use should therefore be viewed as a potentially transitional pattern rather than a harm‐reduction endpoint.

## Graphical abstract

In Scottish Health Survey data, dual users smoked slightly fewer cigarettes than cigarette‐only smokers, but this difference was concentrated among daily e‐cigarette users. Nondaily dual users did not differ from cigarette‐only smokers, suggesting dual use may be transitional but should not be treated as a harm‐reduction endpoint in itself.

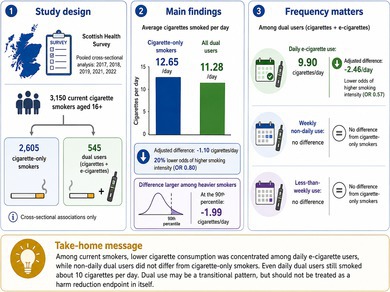

## 1 Introduction

Cigarette smoking remains the leading preventable cause of morbidity and mortality worldwide, responsible for more than seven million deaths annually and imposing a substantial burden on healthcare systems and economies globally [1]. In Scotland, despite sustained declines in smoking prevalence over the past two decades, approximately 13% of adults continued to smoke as of 2025, with particularly high rates concentrated among populations experiencing socioeconomic disadvantage [2]. Reducing the health harms attributable to cigarette smoking therefore remains a central public health priority.

Against this backdrop, electronic cigarettes (e‐cigarettes) have emerged as a widely used nicotine delivery technology, with prevalence increasing markedly across high‐income countries since the early 2010s [3, 4]. E‐cigarettes deliver nicotine through the aerosolisation of a liquid solution rather than through the combustion of tobacco, and the available evidence suggests that the toxicant profile of e‐cigarette aerosol is substantially less harmful than that of cigarette smoke, although not without risk [5, 6]. This differential risk profile has generated considerable interest in the potential role of e‐cigarettes as a harm reduction tool for current smokers who are unable or unwilling to achieve abrupt cessation [7, 8].

A central question in this debate concerns the phenomenon of dual use—the concurrent use of both e‐cigarettes and conventional cigarettes. Dual use is the most common pattern of e‐cigarette use among current smokers, with population‐based surveys consistently reporting that a majority of adult e‐cigarette users continue to smoke cigarettes concurrently [9, 10]. Conceptually, dual use may take two forms: ‘displacement’ dual use, in which e‐cigarettes partially substitute for cigarette consumption, and ‘add‐on’ dual use, in which e‐cigarettes supplement an essentially unchanged level of smoking [11, 12]. The distinction matters because the two patterns carry very different public health implications. Where e‐cigarette use is layered onto unchanged smoking, combustible cigarette exposure continues, and dual use is unlikely to provide the level of harm reduction expected from complete switching [13]. Where displacement occurs, its potential value depends on whether cigarette consumption is meaningfully reduced and whether dual use progresses to complete switching or cessation rather than persisting as a stable pattern [12, 13]. Cross‐sectional data cannot establish which of these patterns predominates, but they can quantify the size of consumption differences associated with dual use and identify the subgroups in which any differences are concentrated.

Existing evidence on the association between dual use and cigarette consumption is mixed and limited in several important respects. A number of cross‐sectional studies have reported that dual users smoke fewer cigarettes per day on average than cigarette‐only smokers [14, 15, 16], whereas others have found no significant difference or have noted that observed differences are attenuated after adjustment for confounders [17, 18]. Longitudinal evidence from the Population Assessment of Tobacco and Health (PATH) Study in the United States and evidence from smoking‐cessation trials suggest that e‐cigarette use may facilitate reductions in cigarette consumption and support eventual cessation [7, 19, 20], although the generalisability of these findings to the broader population of dual users remains uncertain.

A further limitation of the existing literature is the widespread treatment of dual users as a homogeneous group. Dual users vary considerably in their frequency of e‐cigarette use, ranging from occasional or situational use to sustained daily use, and the add‐on versus displacement distinction is plausibly patterned by this frequency [11, 12, 21, 22]. Despite this conceptual distinction, few population‐based studies have examined whether the frequency of e‐cigarette use among dual users is associated with differential levels of cigarette consumption. Quantifying this heterogeneity is a prerequisite for assessing whether, and for whom, dual use is accompanied by reduced smoking, and for informing the design of longitudinal studies capable of addressing the causal question.

The present study addresses these gaps using data from the Scottish Health Survey, a nationally representative household survey with detailed information on both smoking behaviour and e‐cigarette use. Scotland provides a particularly informative setting for this analysis because of its relatively high smoking prevalence, its active policy engagement with tobacco harm reduction and the availability of repeated cross‐sectional data enabling pooled analyses with adequate sample sizes for subgroup comparisons. The primary objective was to estimate the difference in daily cigarette consumption and smoking intensity between current cigarette smokers who also used e‐cigarettes and cigarette‐only smokers, after adjustment for sociodemographic confounders. A secondary objective was to examine whether any difference varied by frequency of e‐cigarette use among dual users. Given the cross‐sectional design, both objectives were explicitly descriptive and association‐focused; no causal effects are claimed.

## 2 Methods

### 2.1 Study Design, Data Source and Study Population

This study was a pooled cross‐sectional analysis of the Scottish Health Survey, combining data from the 2017, 2018, 2019, 2021 and 2022 survey waves. The regular 2020 survey was not included because face‐to‐face fieldwork was suspended owing to the COVID‐19 pandemic, and the separate 2020 telephone survey differed from the standard annual survey in its design and data collection. The Scottish Health Survey is a nationally representative household survey of people living in private households in Scotland, commissioned by the Scottish Government and conducted by the Scottish Centre for Social Research [23]. The survey is designed to provide population‐level information on health, health behaviours and sociodemographic characteristics using standardised questionnaires administered through computer‐assisted personal or telephone interviewing, depending on the survey year [23]. All participants provided informed consent.

The pooled dataset initially included 31 286 respondents across the five survey years. Because the present study focused on adult smoking behaviour, 8925 respondents aged under 16 years were first excluded as they were not eligible for the adult smoking questions, yielding an age‐eligible sample of 22 361 respondents aged 16 years and over. From this sample, 3322 current cigarette smokers were identified and classified into two mutually exclusive groups based on current e‐cigarette use status: 2750 cigarette‐only smokers and 572 dual users. Internal verification confirmed that all respondents in both groups were current cigarette smokers, and that all dual users were concurrently using both cigarettes and e‐cigarettes at the time of survey.

To derive the final analytic sample, 110 respondents with missing or nonsubstantive information on the number of cigarettes smoked per day were excluded, followed by a further 62 current smokers who reported zero average cigarettes per day. The analysis was therefore restricted to current smokers reporting positive average daily cigarette consumption.

### 2.2 Exposure

The main exposure was current e‐cigarette use status among current cigarette smokers. Respondents were classified as cigarette‐only smokers if they currently smoked cigarettes but did not currently use e‐cigarettes, and as dual users if they currently smoked cigarettes and also currently used e‐cigarettes. These two groups formed the basis of all primary comparisons. For the secondary analysis, dual users were further classified by self‐reported frequency of e‐cigarette use in the past 4 weeks (daily, weekly but not daily and less than weekly). The survey does not capture the quantity of e‐cigarette consumption (e.g., puff counts, e‐liquid volume or nicotine concentration); the resulting asymmetry between the measurement of smoking (quantity per day) and e‐cigarette use (frequency categories only) is considered in Section 4.

### 2.3 Outcomes

Two outcome measures were examined. The first was the average number of cigarettes smoked per day, treated as a continuous measure of cigarette consumption. The second was cigarette smoking intensity, classified into three ordered categories: light smoking (fewer than 10 cigarettes per day), moderate smoking (10 to fewer than 20 cigarettes per day) and heavy smoking (20 or more cigarettes per day). These thresholds provide clinically and epidemiologically interpretable consumption categories and are consistent with classifications used in previous research on smoking behaviour [24, 25].

### 2.4 Covariates

Adjusted analyses included a priori sociodemographic covariates selected as plausible confounders of the association between dual‐use status and cigarette consumption [26, 27]. These were age group (seven categories: 16–24, 25–34, 35–44, 45–54, 55–64, 65–74 and 75+), sex (male or female); area‐level deprivation (quintiles of the Scottish Index of Multiple Deprivation), ethnicity (White Scottish vs. all other ethnic groups, owing to small numbers in several minority ethnic categories) and educational attainment (six categories ranging from degree‐level education or higher to no formal qualifications). Survey year was included in all adjusted models to account for secular trends.

### 2.5 Statistical Analysis

Descriptive characteristics of the sample were summarised overall and stratified by e‐cigarette use status. Categorical variables were presented as frequencies and column percentages and compared using Pearson's chi‐square tests. Average cigarettes smoked per day were summarised using means and standard deviations and compared between groups using two‐sample *t*‐tests. Because the distribution of cigarette consumption was positively skewed, medians and interquartile ranges were also reported and compared using the Wilcoxon rank‐sum test.

For the main regression analyses, e‐cigarette use status was treated as the exposure, with cigarette‐only smokers as the reference group. The first outcome, average cigarettes smoked per day, was analysed using linear regression with robust standard errors to account for heteroskedasticity. Three models were estimated: an unadjusted model (Model 1), a model adjusted for age and sex (Model 2) and a fully adjusted model including all covariates and survey year (Model 3). Beta coefficients and 95% confidence intervals were reported, with negative coefficients indicating lower average cigarette consumption among dual users relative to cigarette‐only smokers.

The second outcome, smoking intensity, was modelled using generalised ordered logit regression to accommodate the ordinal nature of the three‐category outcome. The proportional odds assumption was assessed for each covariate, and partial proportional odds models were applied using an autofit approach where the parallel lines constraint was relaxed for variables that violated it. Three sequential models were estimated as above. Odds ratios below 1 indicated lower odds of being in a higher smoking intensity category among dual users compared with cigarette‐only smokers.

To examine whether the association between e‐cigarette use and cigarette consumption varied across the distribution of smoking, quantile regression analyses were conducted for the 25th, 50th, 75th and 90th percentiles of average cigarettes smoked per day, fully adjusted for all covariates and survey year. Quantile regression coefficients were interpreted as differences in cigarette consumption at the specified quantile, comparing dual users with cigarette‐only smokers.

An additional analysis examined whether the association between e‐cigarette use and cigarette consumption outcomes varied according to frequency of use. Current cigarette smokers were classified into four groups: cigarette‐only smokers (reference), less‐than‐weekly e‐cigarette users, weekly nondaily e‐cigarette users and daily e‐cigarette users. This analysis included 3092 respondents after excluding 58 who could not be classified into an active e‐cigarette‐use frequency category: 49 who reported no e‐cigarette use in the past 4 weeks despite being classified as current e‐cigarette users, eight with schedule‐not‐applicable responses and one with a do‐not‐know response. Fully adjusted linear regression and generalised ordered logit models were fitted using the four‐group frequency variable. An omnibus Wald test was used to assess whether cigarette consumption differed across the four groups. Postestimation linear contrasts were then used to compare the adjusted coefficient for daily e‐cigarette use directly with those for weekly nondaily and less‐than‐weekly use, and to compare the two nondaily groups. All analyses used unweighted data [28, 29]. The study was not preregistered, and the analyses should therefore be considered exploratory.

All hypothesis tests were two‐sided, and statistical significance was set at *p* < 0.05. All analyses were conducted using Stata 18 (StataCorp, College Station, TX).

## 3 Results

### 3.1 Sample Characteristics

Table 1 presents the characteristics of the 3150 current cigarette smokers in the analytic sample. Of these, 2605 (82.7%) were cigarette‐only smokers and 545 (17.3%) were dual users of cigarettes and e‐cigarettes. Dual users were younger than cigarette‐only smokers (*p* < 0.001), with a greater proportion aged 35–54 years and a smaller proportion aged 65 years and over. Dual users had higher levels of educational attainment than cigarette‐only smokers (*p* = 0.015), with 19.1% holding a degree or higher qualification compared with 16.5% of cigarette‐only smokers, whereas 20.4% of dual users had no qualifications compared with 25.9% of cigarette‐only smokers. There were no statistically significant differences between the two groups in sex (*p* = 0.162), area deprivation (*p* = 0.426) or ethnicity (*p* = 0.274).

**TABLE 1:** Characteristics of current cigarette smokers by e‐cigarette use status.

Characteristic	Overall (*N* = 3150)	Cigarette‐only smokers (*n* = 2605)	Cigarette smokers who used e‐cigarettes (*n* = 545)	*p*
Age group, *n* (%)				**< 0.001**
16–24	236 (7.5)	185 (7.1)	51 (9.4)	
25–34	506 (16.1)	418 (16.0)	88 (16.2)	
35–44	523 (16.6)	417 (16.0)	106 (19.4)	
45–54	672 (21.3)	540 (20.7)	132 (24.2)	
55–64	607 (19.3)	505 (19.4)	102 (18.7)	
65–74	425 (13.5)	371 (14.2)	54 (9.9)	
75+	181 (5.7)	169 (6.5)	12 (2.2)	
Sex, *n* (%)				**0.162**
Male	1467 (46.6)	1228 (47.1)	239 (43.9)	
Female	1683 (53.4)	1377 (52.9)	306 (56.1)	
Area deprivation, *n* (%)				**0.426**
Least deprived	302 (9.6)	252 (9.7)	50 (9.2)	
4th quintile	469 (14.9)	379 (14.6)	90 (16.5)	
3rd quintile	604 (19.2)	513 (19.7)	91 (16.7)	
2nd quintile	802 (25.5)	656 (25.2)	146 (26.8)	
Most deprived	973 (30.9)	805 (30.9)	168 (30.8)	
Ethnicity, *n* (%)				**0.274**
White Scottish	2602 (82.6)	2143 (82.3)	459 (84.2)	
All other ethnic groups	548 (17.4)	462 (17.7)	86 (15.8)	
Educational attainment, *n* (%)				**0.015**
Degree or higher	535 (17.0)	431 (16.5)	104 (19.1)	
HNC/D or equivalent	400 (12.7)	315 (12.1)	85 (15.6)	
Higher grade or equivalent	513 (16.3)	424 (16.3)	89 (16.3)	
Standard grade or equivalent	760 (24.1)	624 (24.0)	136 (25.0)	
Other school level	157 (5.0)	137 (5.3)	20 (3.7)	
No qualifications	785 (24.9)	674 (25.9)	111 (20.4)	
Smoking intensity, *n* (%)				**0.007**
Light (< 10/day)	1082 (34.4)	870 (33.4)	212 (38.9)	
Moderate (10 to < 20/day)	1348 (42.8)	1115 (42.8)	233 (42.8)	
Heavy (20+/day)	720 (22.9)	620 (23.8)	100 (18.4)	
Cigarettes smoked per day				
Mean (SD)	12.42 (7.81)	12.65 (7.94)	11.28 (7.07)	**0.0002**
Median (IQR)	10.00 (6.57–16.57)	10.71 (7.00–18.00)	10.00 (5.57–15.00)	**0.0004**

*Note:* Values are *n* (%) unless otherwise indicated. Percentages for grouped characteristics are column percentages. *p*‐values for categorical variables were derived from Pearson's chi‐square tests. The *p*‐value for mean cigarettes smoked per day was derived from a two‐sample *t*‐test, and the *p*‐value for median cigarettes smoked per day was derived from the Wilcoxon rank‐sum test.

Dual users smoked, on average, 1.37 fewer cigarettes per day than cigarette‐only smokers, with a mean of 11.28 (SD: 7.07) versus 12.65 (SD: 7.94) cigarettes per day (*p* = 0.0002). The median was also lower among dual users (10.00; IQR: 5.57–15.00) compared with cigarette‐only smokers (10.71; IQR: 7.00–18.00; *p* = 0.0004). The distribution of smoking intensity differed between groups (*p* = 0.007): 38.9% of dual users were light smokers compared with 33.4% of cigarette‐only smokers, whereas 18.4% of dual users were heavy smokers compared with 23.8% of cigarette‐only smokers. Both groups therefore averaged in the region of half a pack of cigarettes per day.

### 3.2 Association Between E‐Cigarette Use and Average Cigarettes Smoked per Day

Table 2 presents the results of linear regression models for average cigarettes smoked per day. In the unadjusted model, dual users smoked 1.37 fewer cigarettes per day than cigarette‐only smokers (β = −1.37; 95% CI: −2.04 to −0.70; *p* < 0.001). This association was modestly attenuated but remained statistically significant after adjustment for age and sex (β = −1.13; 95% CI: −1.77 to −0.49; *p* = 0.001) and after full adjustment for all covariates and survey year (β = −1.10; 95% CI: −1.73 to −0.47; *p* = 0.001).

**TABLE 2:** Association between e‐cigarette use among current cigarette smokers and average cigarettes smoked per day.

Model	Comparison	β (95% CI), *p*
Model 1: Unadjusted	Cigarette smokers who used e‐cigarettes vs. cigarette‐only smokers	−1.37 (−2.04 to −0.70), *p* < 0.001
Model 2: Age‐ and sex‐adjusted	Cigarette smokers who used e‐cigarettes vs. cigarette‐only smokers	−1.13 (−1.77 to −0.49), *p* = 0.001
Model 3: Fully adjusted	Cigarette smokers who used e‐cigarettes vs. cigarette‐only smokers	−1.10 (−1.73 to −0.47), *p* = 0.001

*Note:* Beta coefficients represent the difference in average cigarettes smoked per day for cigarette smokers who used e‐cigarettes relative to cigarette‐only smokers. Negative coefficients indicate lower consumption. Model 1 was unadjusted. Model 2 adjusted for age and sex. Model 3 adjusted for age, sex, area deprivation, ethnicity, educational attainment and survey year.

### 3.3 Association Between E‐Cigarette Use and Smoking Intensity Category

Table 3 presents the results of generalised ordered logit models for smoking intensity, classified as light smoking (< 10 cigarettes/day), moderate smoking (10 to < 20/day) and heavy smoking (20+/day). In the unadjusted model, dual users had 24% lower odds of being in a heavier smoking category rather than a lighter one compared with cigarette‐only smokers (OR = 0.76; 95% CI: 0.64 to 0.91; *p* = 0.002). After full adjustment, dual users remained less likely than cigarette‐only smokers to be in a heavier smoking category (OR = 0.80; 95% CI: 0.67 to 0.96; *p* = 0.014).

**TABLE 3:** Association between e‐cigarette use among current cigarette smokers and smoking intensity category.

Model	Comparison	OR (95% CI), *p*
Model 1: Unadjusted	Cigarette smokers who used e‐cigarettes vs. cigarette‐only smokers	0.76 (0.64 to 0.91), *p* = 0.002
Model 2: Age‐ and sex‐adjusted	Cigarette smokers who used e‐cigarettes vs. cigarette‐only smokers	0.80 (0.67 to 0.95), *p* = 0.011
Model 3: Fully adjusted	Cigarette smokers who used e‐cigarettes vs. cigarette‐only smokers	0.80 (0.67 to 0.96), *p* = 0.014

*Note:* Odds ratios were estimated using generalised ordered logit models. The outcome was smoking intensity classified as light (< 10 cigarettes/day), moderate (10 to < 20/day) and heavy (20+/day). Odds ratios below 1 indicate lower odds of being in a heavier smoking category rather than a lighter one among cigarette smokers who used e‐cigarettes compared with cigarette‐only smokers. Model 1 was unadjusted. Model 2 adjusted for age and sex. Model 3 adjusted for age, sex, area deprivation, ethnicity, educational attainment and survey year.

### 3.4 Quantile Regression Analysis

**FIGURE 1:**
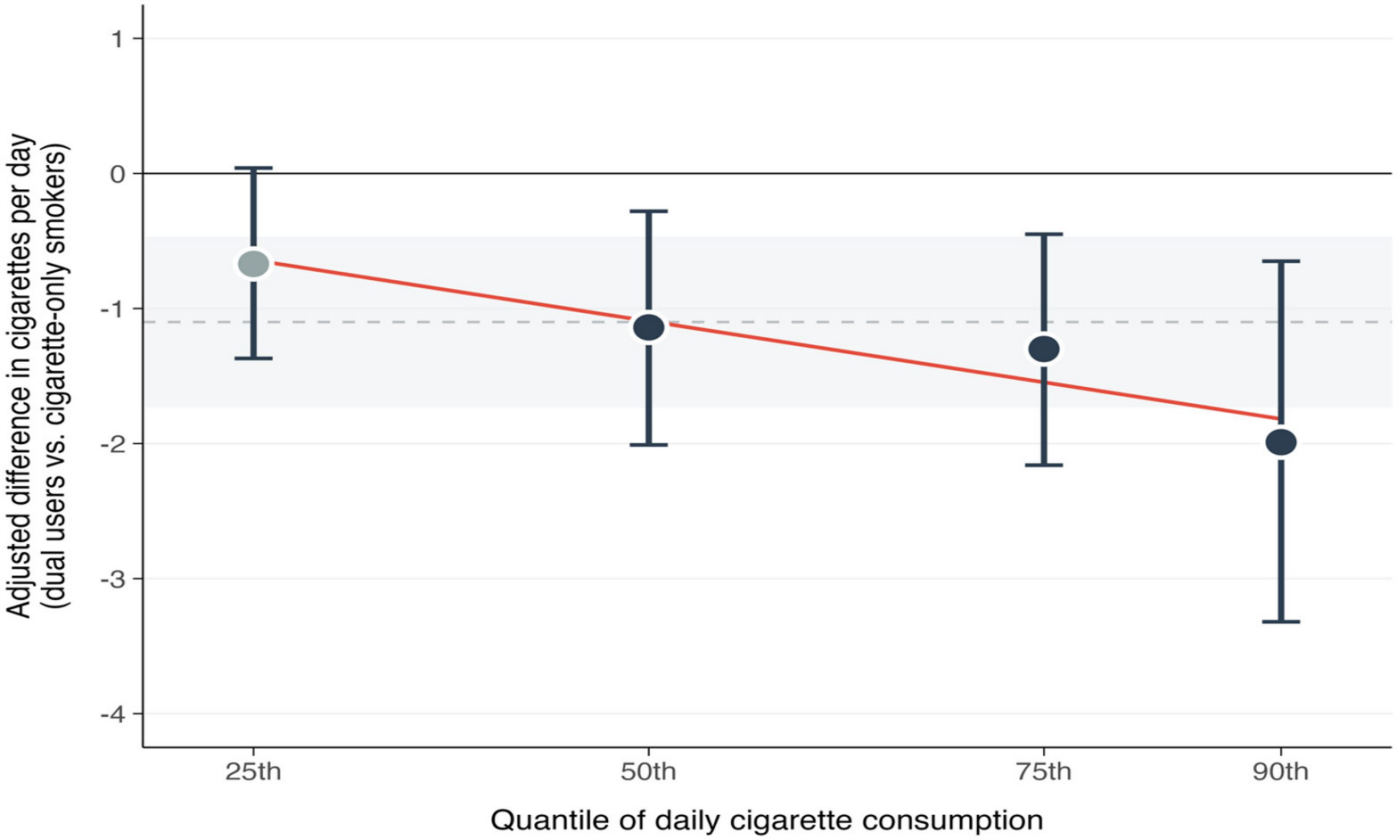
Adjusted quantile regression coefficients for the association between e‐cigarette use and daily cigarette consumption among current smokers. Points represent adjusted differences in cigarettes smoked per day comparing dual users with cigarette‐only smokers at the 25th, 50th, 75th and 90th percentiles; vertical bars indicate 95% confidence intervals. The dashed horizontal line represents the fully adjusted mean difference estimated using linear regression, and the shaded area represents its 95% confidence interval. The connecting line is included to illustrate the pattern across quantiles.

Table 4 and Figure 1 present fully adjusted quantile regression estimates for cigarettes smoked per day. The association between e‐cigarette use and cigarettes smoked per day was not statistically significant at the 25th percentile of the distribution (β = −0.67; 95% CI: −1.37 to 0.04; *p* = 0.063), but was statistically significant and progressively larger at the 50th percentile (β = −1.14; 95% CI: −2.01 to −0.28; *p* = 0.009), 75th percentile (β = −1.30; 95% CI: −2.16 to −0.45; *p* = 0.003) and 90th percentile (β = −1.99; 95% CI: −3.32 to −0.65; *p* = 0.004). The inverse association between e‐cigarette use and cigarettes smoked per day was therefore more evident among smokers at the higher end of the consumption distribution, although the largest estimated difference remained below two cigarettes per day.

**TABLE 4:** Quantile regression of average cigarettes smoked per day by e‐cigarette use status.

Quantile	Comparison	Adjusted β (95% CI), *p*
25th percentile	Cigarette smokers who used e‐cigarettes vs. cigarette‐only smokers	−0.67 (−1.37 to 0.04), *p* = 0.063
50th percentile	Cigarette smokers who used e‐cigarettes vs. cigarette‐only smokers	−1.14 (−2.01 to −0.28), *p* = 0.009
75th percentile	Cigarette smokers who used e‐cigarettes vs. cigarette‐only smokers	−1.30 (−2.16 to −0.45), *p* = 0.003
90th percentile	Cigarette smokers who used e‐cigarettes vs. cigarette‐only smokers	−1.99 (−3.32 to −0.65), *p* = 0.004

*Note:* Quantile regression models were adjusted for age, sex, area deprivation, ethnicity, educational attainment and survey year. Beta coefficients represent the difference in cigarettes smoked per day among cigarette smokers who used e‐cigarettes relative to cigarette‐only smokers at the specified quantile of the cigarettes‐per‐day distribution.

### 3.5 E‐Cigarette Use Frequency and Cigarette Consumption

Table 5 presents the results of the frequency analysis among 3092 current cigarette smokers. The association between concurrent e‐cigarette use and lower cigarette consumption was confined to daily e‐cigarette users. In fully adjusted models, daily e‐cigarette users smoked 2.46 fewer cigarettes per day than cigarette‐only smokers (mean: 9.90 vs. 12.65; β = −2.46; 95% CI: −3.32 to −1.59; *p* < 0.001) and had 43% lower odds of being in a higher smoking‐intensity category (OR = 0.57; 95% CI: 0.44 to 0.74; *p* < 0.001). By contrast, neither less‐than‐weekly e‐cigarette users (β = −0.24; *p* = 0.751) nor weekly nondaily e‐cigarette users (β = −0.23; *p* = 0.661) differed from cigarette‐only smokers in average cigarettes smoked per day. The corresponding odds ratios for smoking intensity were similarly close to the null (less‐than‐weekly: OR = 0.98; *p* = 0.924; weekly nondaily: OR = 1.03; *p* = 0.830). Cigarette consumption differed overall across the four frequency groups, *F*(3, 3071) = 10.32, *p* < 0.001. Formal contrasts showed that daily e‐cigarette users smoked 2.21 fewer cigarettes per day than less‐than‐weekly users (95% CI: −3.90 to −0.52; *p* = 0.010) and 2.23 fewer cigarettes per day than weekly nondaily users (95% CI: −3.50 to −0.96; *p* = 0.001). Weekly nondaily and less‐than‐weekly users did not differ from one another (difference = 0.02 cigarettes per day; 95% CI: −1.75 to 1.79; *p* = 0.985). There was therefore no evidence of a graded association across the nondaily frequency categories: Estimates for both nondaily groups were close to the null, and lower cigarette consumption was observed only among daily e‐cigarette users.

**TABLE 5:** E‐cigarette use frequency among current cigarette smokers and cigarette consumption outcomes.

E‐cigarette use frequency group	*n*	Mean cigarettes/day (SD)	Median (IQR)	Adjusted β for cigarettes/day (95% CI), *p* ^a^	Adjusted OR for higher smoking‐intensity category (95% CI), *p* ^b^
Cigarette‐only smokers	2605	12.65 (7.94)	10.71 (7.00–18.00)	Reference	Reference
Less‐than‐weekly e‐cigarette use	67	11.91 (7.00)	10.00 (7.71–15.00)	−0.24 (−1.76 to 1.27), *p* = 0.751	0.98 (0.62 to 1.53), *p* = 0.924
Weekly nondaily e‐cigarette use	185	12.22 (7.06)	10.00 (7.00–15.00)	−0.23 (−1.25 to 0.79), *p* = 0.661	1.03 (0.78 to 1.37), *p* = 0.830
Daily e‐cigarette use	235	9.90 (6.81)	10.00 (4.00–15.00)	−2.46 (−3.32 to −1.59), *p* < 0.001	0.57 (0.44 to 0.74), *p* < 0.001

*Note:* This analysis was based on 3092 current cigarette smokers. Starting from the final analytic sample of 3150, we excluded 58 respondents who could not be classified into an active e‐cigarette use frequency category, including 49 who reported no e‐cigarette use in the past 4 weeks, eight with schedule‐not‐applicable responses and one with a do‐not‐know response.

^a^ Adjusted beta coefficients were estimated from linear regression models for average cigarettes smoked per day, controlling for age, sex, area deprivation, ethnicity, educational attainment and survey year. Negative coefficients indicate lower cigarette consumption relative to cigarette‐only smokers.

^b^ Adjusted odds ratios were estimated from generalised ordered logit models for smoking intensity category, controlling for age, sex, area deprivation, ethnicity, educational attainment and year. Odds ratios below 1 indicate lower odds of being in a higher smoking‐intensity category relative to cigarette‐only smokers.

## 4 Discussion

This pooled cross‐sectional analysis of 3150 current cigarette smokers from the Scottish Health Survey found that concurrent e‐cigarette use was associated with lower daily cigarette consumption and lower odds of occupying a higher smoking‐intensity category, although the absolute differences were modest. In fully adjusted models, dual users smoked approximately one fewer cigarette per day than cigarette‐only smokers (11.28 vs. 12.65 cigarettes per day on average) and had 20% lower odds of being in a higher smoking‐intensity category. When dual users were stratified by frequency of e‐cigarette use, the association was confined to daily e‐cigarette users, who smoked, on average, 2.46 fewer cigarettes per day than cigarette‐only smokers (9.90 vs. 12.65). Nondaily e‐cigarette use, whether weekly or less‐than‐weekly, was not associated with lower cigarette consumption. Two features of these results should be stated plainly. First, even daily dual users smoked approximately half a pack of cigarettes per day on average. Second, among the majority of classifiable dual users who reported nondaily e‐cigarette use, 252 of 487 (51.7%), e‐cigarette use was not associated with lower cigarette consumption and was therefore more consistent with supplementary than displacement use.

The magnitude of these differences warrants careful interpretation. A difference of approximately one cigarette per day in the pooled comparison, or two and a half cigarettes per day among daily e‐cigarette users, should not be assumed to translate into a proportionate individual health benefit. The dose–response relationship between cigarette consumption and disease risk is markedly non‐linear: Meta‐analytic evidence indicates that smoking even one to five cigarettes per day carries a substantial proportion of the cardiovascular risk associated with heavier smoking, such that reductions in consumption short of complete cessation confer far smaller risk reductions than might be expected from a proportional relationship [30]. Although a reduction of approximately two and a half cigarettes per day may represent behavioural progress for some smokers, daily e‐cigarette users still smoked about 10 cigarettes per day on average. The appropriate interpretation is therefore not that dual use, as a category, confers meaningful harm reduction, but that lower cigarette consumption associated with dual use was modest and concentrated among daily e‐cigarette users.

The finding that dual users smoked slightly fewer cigarettes per day than cigarette‐only smokers is broadly consistent with previous population‐based evidence [14, 31]. The quantile regression findings add a dimension that has received limited attention in earlier research: The inverse association was larger at the upper end of the cigarette‐consumption distribution, reaching approximately two fewer cigarettes per day at the 90th percentile. This pattern is compatible with the possibility that differences associated with dual use are more pronounced among heavier smokers. A similar pattern has been reported in analyses of the PATH study, in which dual users with higher levels of e‐cigarette use showed greater reductions in cigarette consumption [32]. However, the present pattern could also reflect residual confounding, selection or greater heterogeneity in smoking behaviour among heavier smokers and should therefore be interpreted as descriptive.

The most notable finding of the present study was the clear distinction between daily and nondaily e‐cigarette use. This pattern aligns with the conceptual distinction between displacement and add‐on dual use proposed by Kroeger and colleagues [11]. Viewed through this framework, nondaily dual use, the majority pattern in this sample, was more consistent with supplementary use, because it was not associated with lower cigarette consumption, whereas daily use was the only frequency pattern associated with lower consumption. One plausible explanation is that daily e‐cigarette use provides sufficiently regular nicotine substitution to replace some smoking occasions, whereas nondaily use may be more situational and therefore less likely to alter usual cigarette consumption. However, the association may also reflect selection or reverse causation: Smokers who are lighter, more motivated to reduce or quit or already engaged in a quit attempt may be more likely to adopt and sustain daily e‐cigarette use. The distinction between daily and nondaily use may therefore reflect partial cigarette displacement, differences in smoker characteristics, or a combination of both. The cross‐sectional design cannot distinguish among these explanations. Interpretation should also remain cautious because e‐cigarette exposure was measured using broad frequency categories rather than quantity or nicotine intake.

From a public health perspective, the principal implication of these findings is cautionary but compatible with a harm‐reduction framework. Dual use is not a homogeneous pattern, and its potential value depends on whether e‐cigarette use meaningfully displaces combustible cigarette consumption or supports eventual complete switching. In the present study, dual use was not associated with substantively lower cigarette consumption among most dual users. Persistent dual use accompanied by largely unchanged cigarette consumption is unlikely to provide the level of harm reduction expected from complete switching because combustible cigarette smoking continues alongside e‐cigarette use; this interpretation is consistent with systematic‐review evidence showing no clear demonstrated health benefit of dual use relative to exclusive cigarette smoking [13]. Dual use should therefore not be regarded as a harm‐reduction endpoint in itself. Rather, it may be understood as a potentially transitional pattern whose value depends on whether it leads to complete switching, smoking cessation or sustained and substantial reductions in cigarette consumption. Evidence from randomised trials supports the use of e‐cigarettes as a route to smoking cessation or complete substitution among adults who smoke [7]. The present findings further suggest that infrequent or situational e‐cigarette use among continuing smokers is not associated with the modestly lower cigarette consumption observed among daily users.

This study has several strengths. The use of a large national household survey with standardised data‐collection protocols enhances the relevance of the findings to community‐dwelling adults who smoke in Scotland. Pooling five survey waves produced an analytic sample of more than 3000 current smokers and enabled frequency‐specific subgroup analyses, although estimates were less precise for the less‐than‐weekly group. The analytical approach was comprehensive, combining linear regression, generalised ordered logit models and quantile regression to characterise associations across different dimensions of cigarette consumption. The frequency‐specific analysis revealed important heterogeneity within the dual‐user population that would have been obscured by treating dual use as a single category.

Several limitations warrant consideration. First, the cross‐sectional design does not permit causal inference regarding the direction or mechanisms of the observed associations. Longitudinal data would be required to determine whether e‐cigarette adoption precedes lower cigarette consumption or whether pre‐existing smoking patterns, quit motivation or other smoker characteristics predict e‐cigarette uptake and frequency of use. Second, there was an important asymmetry in how the two behaviours were measured: Cigarette smoking was quantified as cigarettes per day, whereas e‐cigarette use was captured only through broad frequency categories, daily, weekly nondaily and less‐than‐weekly, with no information on quantity, device type, e‐liquid nicotine concentration or puffing intensity. This limited the examination of dose–response patterns within the daily‐use group, where actual e‐cigarette consumption and nicotine intake may vary substantially, and introduced exposure measurement imprecision. Consequently, the apparent distinction between daily and nondaily use may partly reflect the coarseness of the exposure measure rather than a true behavioural threshold.

Third, all measures of cigarette and e‐cigarette use were self‐reported and may have been affected by recall or social‐desirability bias; biochemical validation was unavailable. Fourth, cigarette smoking was examined in terms of cigarettes per day and smoking‐intensity category only. Smoking frequency, such as the number of days smoked per month or everyday versus nondaily smoking, was not examined and may show different associations. Future studies using datasets that capture smoking days should examine frequency alongside quantity. Fifth, the less‐than‐weekly e‐cigarette‐use group was small (*n* = 67), resulting in imprecise estimates and limited ability to detect modest differences. Although the weekly nondaily group was larger (*n* = 185), its estimate was also close to the null. Sixth, information on reasons for e‐cigarette use, nicotine dependence, prior quit attempts and current intention to reduce or stop smoking was unavailable. Residual confounding by these factors cannot therefore be excluded, and differences in quit motivation remain a plausible explanation for the observed frequency‐specific associations.

Finally, the analyses were conducted without survey weights. While the regression models adjusted for the principal demographic and socioeconomic variables upon which the Scottish Health Survey weights are calibrated, it remains possible that residual features of the complex survey design, such as clustering within primary sampling units, may affect standard error estimates. However, the focus of this study on internal associations within a restricted subpopulation, rather than on population‐level prevalence estimation, may limit the effect of not applying survey weights [28, 29].

## 5 Conclusions

Among current cigarette smokers in Scotland, concurrent e‐cigarette use was associated with modestly lower daily cigarette consumption, averaging approximately one fewer cigarette per day overall and two and a half fewer cigarettes per day among daily e‐cigarette users. No comparable association was observed among nondaily users. Even daily dual users smoked approximately 10 cigarettes per day, or around half a pack, on average. These findings document important heterogeneity within the dual‐user population according to frequency of e‐cigarette use, but they do not establish that dual use produces substantial reductions in smoking‐related health risk. The observed pattern is compatible with partial cigarette displacement among daily users and with supplementary rather than displacement use among many nondaily users. Dual use should therefore be regarded as a potentially transitional pattern whose value depends on whether it leads to complete switching or smoking cessation, rather than as a harm‐reduction endpoint in itself. Future longitudinal research should quantify both cigarette and e‐cigarette consumption, examine smoking frequency as well as quantity, measure quit motivation and nicotine dependence and incorporate biochemical validation to determine whether, for whom and under what patterns of use e‐cigarettes displace rather than accompany cigarette smoking.

## Author Contributions

**Yusuff Adebayo Adebisi:** conceptualisation, methodology, data curation, formal analysis, interpretation of findings, writing – original draft, writing – review and editing and final approval of the manuscript.

## Funding

The author has nothing to report.

## Ethics Statement

This study used anonymised secondary data from the Scottish Health Survey. Ethical approval for the original Scottish Health Survey data collection was obtained by the survey administrators, and participants provided informed consent at the time of data collection. The present study involved secondary analysis of de‐identified data and did not require additional ethical approval or separate informed consent.

## Conflicts of Interest

Yusuff Adebayo Adebisi has previously received funding through the Tobacco Harm Reduction Scholarship and the Kevin Molloy Fellowship, both awarded by Knowledge‐Action‐Change (KAC), a public health organisation based in the United Kingdom. KAC receives funding from Global Action to End Smoking (GA), a US‐based nonprofit grant‐making organisation. These fellowships were not awarded specifically for the present study. The funders had no role in the study design, data analysis, interpretation of findings, manuscript preparation or decision to submit the article for publication.

## Data Availability

The data used in this study are available from the UK Data Service through the Scottish Health Survey data series, subject to registration and the applicable End User Licence conditions. The analytic code used for this study is available from the author upon reasonable request.
